# Cell mass-dependent expression of an anticancer protein drug by tumor-targeted *Salmonella*

**DOI:** 10.18632/oncotarget.24013

**Published:** 2018-01-05

**Authors:** Kwangsoo Kim, Sa-Young Min, Ho-Dong Lim, Sung-Hwan You, Daejin Lim, Jae-Ho Jeong, Hyun-Ju Kim, Joon Haeng Rhee, Kyeongil Park, Minsang Shin, Geun-Joong Kim, Jung-Joon Min, Hyon E. Choy

**Affiliations:** ^1^ Department of Microbiology, Chonnam National University Medical School, Jeollanam-do, Republic of Korea; ^2^ Molecular Medicine, BK21 plus, Chonnam National University Graduate School, Gwangju, Republic of Korea; ^3^ Department of Nuclear Medicine, Chonnam National University Medical School and Hwasun Hospital, Jeonnam, Republic of Korea; ^4^ Department of Biological Sciences, College of Natural Sciences, Chonnam National University, Gwangju, Republic of Korea; ^5^ Department of Microbiology, Kyungpook National University Medical School, Daegu, Republic of Korea

**Keywords:** bacterial cancer therapy, salmonella, quorum-sensing system, L-asparaginase

## Abstract

Bacterial cancer therapy relies on the properties of certain bacterial species capable of targeting and proliferating within solid malignancies. If these bacteria could be loaded with antitumor proteins, the efficacy of this approach could be greatly increased. However, because most antitumor proteins are also toxic to normal tissue, they must be expressed by bacteria that specifically target and exclusively localize to tumor tissue. As a strategy for treating solid malignancies, we recently evaluated L-asparaginase (L-ASNase) delivered by tumor-targeted *Salmonella*. In this system, L-ASNase was expressed under the control of the *araBAD* promoter (*PBAD*) of the *E. coli* arabinose operon, which is induced by injection of L-arabinose. Here, we further improved the performance of recombinant *Salmonella* in cancer therapy by exploiting the quorum-sensing (QS) system, which uses cell mass-dependent auto-induction logic. This approach obviates the necessity of monitoring intratumoral bacterial status and inducing cargo protein expression by administration of an exogenous compound. Recombinant *Salmonella* in tumors expressed and secreted active L-ASNase in a cell mass-dependent manner, yielding significant anticancer effects. These results suggest that expression of a therapeutic protein under the control of the QS system represents a promising engineering platform for the production of recombinant proteins *in vivo*.

## INTRODUCTION

Bacterial cancer therapy relies on the natural propensity of certain bacteria, including *Escherichia coli* [1], *Salmonella* [2], *Clostridium* [3], and *Listeria* [4], to accumulate and replicate in various types of tumors [5]. However, the strains must be modified to eliminate factors responsible for bacteria-related toxicity, i.e., products of virulence genes and obvious endotoxins. In the case of *Salmonella typhimurium*, the first such strain (VNP20009) used for cancer therapy carried a mutation in *msbB*, which altered lipid A structure, and *purI*, to confer purine auxotrophy [6–8]. Another strain used in this context is the A1-R mutant, a leucine–arginine auxotroph with elevated antitumor activity that arose during selection by tumor passage [9–14]. This strain exerts extraordinary antitumor effects in patient-derived orthotopic xenograft mouse models, especially in combination with chemotherapy [15–24]. This feature is ascribed to the ability of this strain to induce quiescent cancer cells to cycle, rendering them chemosensitive [25, 26].

In our studies, we have used the ΔppGpp strain of *Salmonella typhimurium*, which lacks both genes encoding ppGpp synthesis enzyme, *relA* and *spot* [27–29], because this strain is capable of targeting all solid tumors tested but is almost a million-fold less virulent than the wild-type strain due to defective expression of the genes encoded by *Salmonella* pathogenicity islands [30, 31]. Recent studies have attributed tumor suppression by ΔppGpp *Salmonella* to a marked increase in production of proinflammatory cytokines, namely, TNF-a and IL-1b, by macrophages and dendritic cells infiltrated into the tumor mass [32, 33].

Although tumor targeting by bacteria alone results in a reduction of tumor mass [5, 9, 34–37], bacteria expressing therapeutic proteins are clearly more efficacious [30, 38, 39]. Because most anticancer proteins are to some degree also toxic to normal cells, their expression must be confined to tumor tissue. Following intravenous (*i.v.*) injection in mice, *Salmonella* rapidly accumulate in reticuloendothelial (RE) systems, liver, and spleen, but to a lesser extent in tumor tissue [38, 39]. Several days later, the *Salmonella* gradually clear from the RE systems but increase in number in tumor tissue at a rate that depends on the strain. This accumulation of *Salmonella* is due in part to the immune-suppressive environment in tumor tissue, which allows proliferation of intratumoral *Salmonella* to densities greater than 10^9^ colony-forming units (CFU)/g of tissue [40, 41]. Using ΔppGpp *Salmonella*, we observed that controlled expression of cytotoxic proteins such as cytolysin [42] or Noxa [43] 3 days post-infection (dpi) did not cause notable systemic toxicity.

L-asparaginase (L-ASNase, EC2) of *E. coli* origin is widely used in therapy of acute lymphoblastic leukemia (ALL) [44]. L-ASNase primarily catalyzes the deamination of asparagine to aspartate, and to a lesser extent the conversion of glutamine to glutamate [45]. In tumor cells defective in asparagine synthetase, depletion of asparagine leads to inhibition of global protein synthesis [46] due to a failure to resupply asparagine [47, 48], resulting in apoptotic cell death. L-ASNase administered via the intravenous (i.v.) route [47] is efficacious against ALL, but not against solid tumors, due to the limited distribution of the drug: L-ASNase injected i.v. rarely accumulates in grafted solid tumors in mouse models [49, 50]. Moreover, i.v. administration of high doses of L-ASNase to tumor-bearing mice results in morbidity and mortality, and the side effects associated with L-ASNase include anaphylactic shock, coagulopathies, and hepatic and pancreatic toxicity [50, 51].

Previously, we engineered *Salmonella* to express L-ASNase selectively within solid tumors using a remote gene control system based on the *araBAD* promoter (*P*BAD) of *E. coli*, which can be induced by intraperitoneal (i.p.) administration of L-arabinose. In mice harboring grafts of solid tumors, we demonstrated the antitumor efficacy of targeted L-ASNase using *Salmonella* transformed with this construct. Daily administration of L-arabinose was required to maintain continuous expression of L-ASNase. However, remote gene control via diffusion from the injection site had some intrinsic problems, including off-target effects and inhomogeneity. Frequent injection was also stressful for the animal. These inherent limitations of the *P*BAD-induced L-ASNase system mandated the development of a system in which L-ASNase is induced in a cell mass-dependent manner. To this end, we turned to a bacterial auto-induction system.

Certain species of bacteria exhibit cooperative behavioral patterns mediated by an environmental sensing mechanism termed the auto-induction system. This system allows bacteria to monitor their own population density by producing, secreting, and sensing a diffusible compound called the auto-inducer, whose concentration in the environment correlates well with local cell density during growth. At low cell densities, the auto-inducer is present at low concentrations, whereas at high cell densities it accumulates to the critical concentration required for activation of its target genes. For example, luminescence in the marine bacteria *Vibrio fischeri* and *Vibrio harveyi* is auto-induced by acyl-homoserine lactone (AHL). In this quorum-sensing (QS) system, the LuxI and LuxR proteins control expression of the luciferase operon (*luxICDABE*), which encodes the proteins required for luminescence. LuxI catalyzes the synthesis of the auto-inducer AHL [52, 53], whereas LuxR is the cytoplasmic AHL receptor that functions as a transcriptional activator upon ligand binding [54]. After synthesis *in vivo*, AHL freely diffuses in and out of bacterial cells, and its concentration increases with local cell density [55]. When AHL reaches a critical threshold concentration, the LuxR-AHL complex activates transcription of the operon encoding luciferase [56].

In this study, we engineered *S. typhimurium* harboring an auto-inducible recombinant plasmid expressing a gene encoding L-ASNase under the control of the *Vibrio* QS expression system, and demonstrated the bacterial density-dependent expression of L-ASNase and its antitumor effects in a mouse graft model.

## RESULTS

### Expression of reporter protein under the control of the QS promoter system

**Figure 1:**
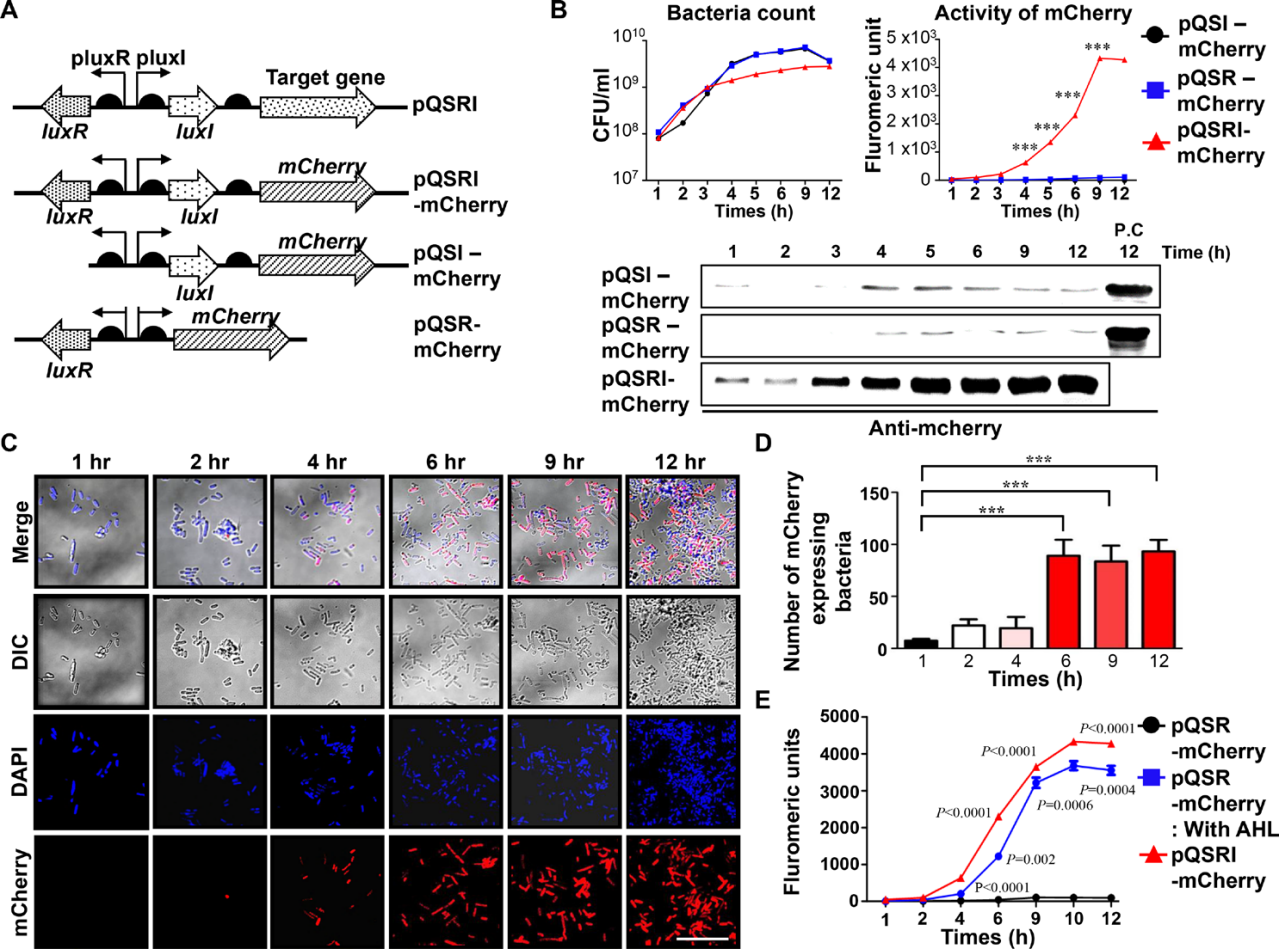
Expression of mCherry reporter gene in ∆ppGpp Salmonella carrying pQSRI-mCherry, pQSI-mCherry, or pQSR-mCherry during growth *in vitro* (**A**) Plasmid constructs carrying mCherry under control of various combinations of QS elements, LuxR, and/or LuxI. (**B**) Bacterial growth (CFU/ml) in LB as a function of time (hours, h) (left panel). Expression of mCherry from bacteria grown in LB, as determined by fluorometry (right panel) and by Western blotting (bottom panel). For detection of mcherry, we used 20µg of purified protein for each sample. Positive control (P.C) is the sample of PQSRI-mcherry (12 hr). (**C**) *Salmonella* carrying the pQSRI-mCherry, grown in LB as shown in (Figure 1B, left panel), were sampled at the indicated time, stained, and imaged under a fluorescence microscope. Blue indicates DAPI staining and red indicates mCherry. Differential interference contrast (DIC) microscopic images of *Salmonella* are included. Scale bar, 10 µm. (**D**) mCherry-expressing *Salmonella* were quantitated ( > 5 fields per sample) visually and plotted as a function of culture time. (**E**) Expression of mCherry by ∆ppGpp *Salmonella* carrying pQSRI-mCherry or pQSR-mCherry supplemented with or without AHL in LB, as determined by fluorometry. *Salmonella* carrying pQSR-mCherry were included as a control. Asterisks (^*^) in panels B and D indicate significant differences compared to pQSI-mCherry (^***^P < 0.0001).

The QS system (*luxR*, *luxI*, and *luxR–luxI* intergenic sequence including the *luxR* and *luxI* promoter) used in this study was deduced from the whole genome sequence of *Vibrio fischeri* and specifically amplified by PCR using primers listed in Table 1. The resultant DNA fragments were assembled by overlapping PCR to exclude incorporation of additional bases, which occurs during typical subcloning procedures using restriction enzyme recognition sites. For efficient co-expression of transcriptionally coupled proteins (mCherry and L-ASNase) with LuxI, a well-known ribosome-binding sequence (RBS) from pQE30 was also included (Figure 1A). mCherry was deployed as a reporter to test the QS elements. Using this method, we constructed three different plasmids carrying the cell density-dependent expression system. pQSRI-mCherry consisted of *luxR* and the *luxR–luxI* intergenic sequence, including both promoters and mCherry transcriptionally coupled with *luxI*. pQSI-mCherry was the same except that it lacked *luxR.* pQSR-mCherry carried the mCherry gene under the direct control of *luxI* promoter, and thus lacked *luxI*, but retained intact *luxR*. The *glmS* gene was incorporated into these recombinant plasmids to generate a balanced-lethal host vector system [57] that complemented the phenotype of the GlmS^-^ mutant, which undergoes lysis unless its catalytic product N-acetyl-D-glucosamine is provided or complemented by a GlmS^+^-containing plasmid. These plasmids were transformed into an avirulent ΔppGpp *Salmonella typhimurium*, 14028s.

**Table 1:** List of primer sequences used in this study

Primer	Sequence(5′-3′)	Gene amplified
LuxRF	CGC**GGATCC**TTAATTTTTAAAGTATGGGCAATCAATTGC	*luxR*
LuxROLR	AAGGATAAAGAGATGGGTATGAAAGACATAAATGCC	
LuxRIproF	CGC**GGATCC**CTCTTTATCCTTTATCCTTACCTATTG	*luxR-luxI* intergenic sequence
LuxRIproOLF	TTTCATACCCATCTCTTTATCCTTTATCCTTACCTATTG	
LuxIR	CTTGCTCACCATAGCTGTCCTCCTTTTTAATTTAAGACTGCTTTTTTAAACTGTTC	*luxI*
LuxIproR	GAAAAACTCCATACCAACCTCCCTTGCGTTTATTC	*luxR-luxI* intergenic sequence
mChOLF	GTCTTAAATTAAAAAGGAGGACAGCTATGGTGAGCAAGGGCGAG	mCherry gene
mChOL-RI F	AGGGAGGTTGGTATGGTGAGCAAGGGCGAG	
mChR	CGC**GTCGAC**TTACTACTTGTACAGCTCGTCC	
ASNOLF	GTCTTAAATTAAAAAGGAGGACAGCTATGGAGTTTTTCAAAAAGACGGCAC	L-Aspragenase gene
ASNOL-RIF	AGGGAGGTTGGTATGGAGTTTTTCAAAAAGACGGCAC	
ASNR	CGC**GTCGAC**TTAGTACTGATTGAAGATCTGCTG	
glmSF	CC**ATCGAT**ATGTGTGGAATTGTTGGC	*glmS*
glmSR	CC**ATCGAT**TTACTCTACGGTAACCGATT	

Restriction sites for *BamH*I (GGATCC), *Sal*I (GTCGAC) and *Cla*I (ATCGAT) are indicated in bold.

*Salmonella* carrying these plasmids were cultured in Luria-Bertani (LB) media, and samples were collected at the indicated times (h) to determine viable cell count (CFU, Figure 1B, left panel) for analysis of mCherry expression by fluorometry (Figure 1B, right panel) and for Western blotting using mCherry-specific antibody (Figure 1B, bottom panel). The results revealed that *Salmonella* carrying pQSRI-mCherry began to show fluorescence at ∼3 h, which saturated at 9 h as the culture density rose above 10^9^ CFU/ml, whereas those carrying pQSI-mCherry or pQSR-mCherry expressed no fluorescence. The expression of mCherry by *Salmonella* carrying pQSRI-mCherry was visualized by fluorescence microscopy (Figure 1C). Bacterial cells expressing mCherry fluorescence began to be detected starting at ∼4 h and increased in number as time passed. Figure 1D shows quantitation of the fluorescent *Salmonella* shown in Figure 1C. *Salmonella* carrying pQSR-mCherry failed to express mCherry because they lacked LuxI and were thus unable to produce auto-inducer [52]. Accordingly, addition of AHL to the media restored mCherry expression (Figure 1E), albeit only partially, as expected. Taken together, these observations demonstrate that a cargo gene transcriptionally coupled with *luxI* in the presence of LuxR can be expressed in a cell density-dependent manner.

**Figure 2:**
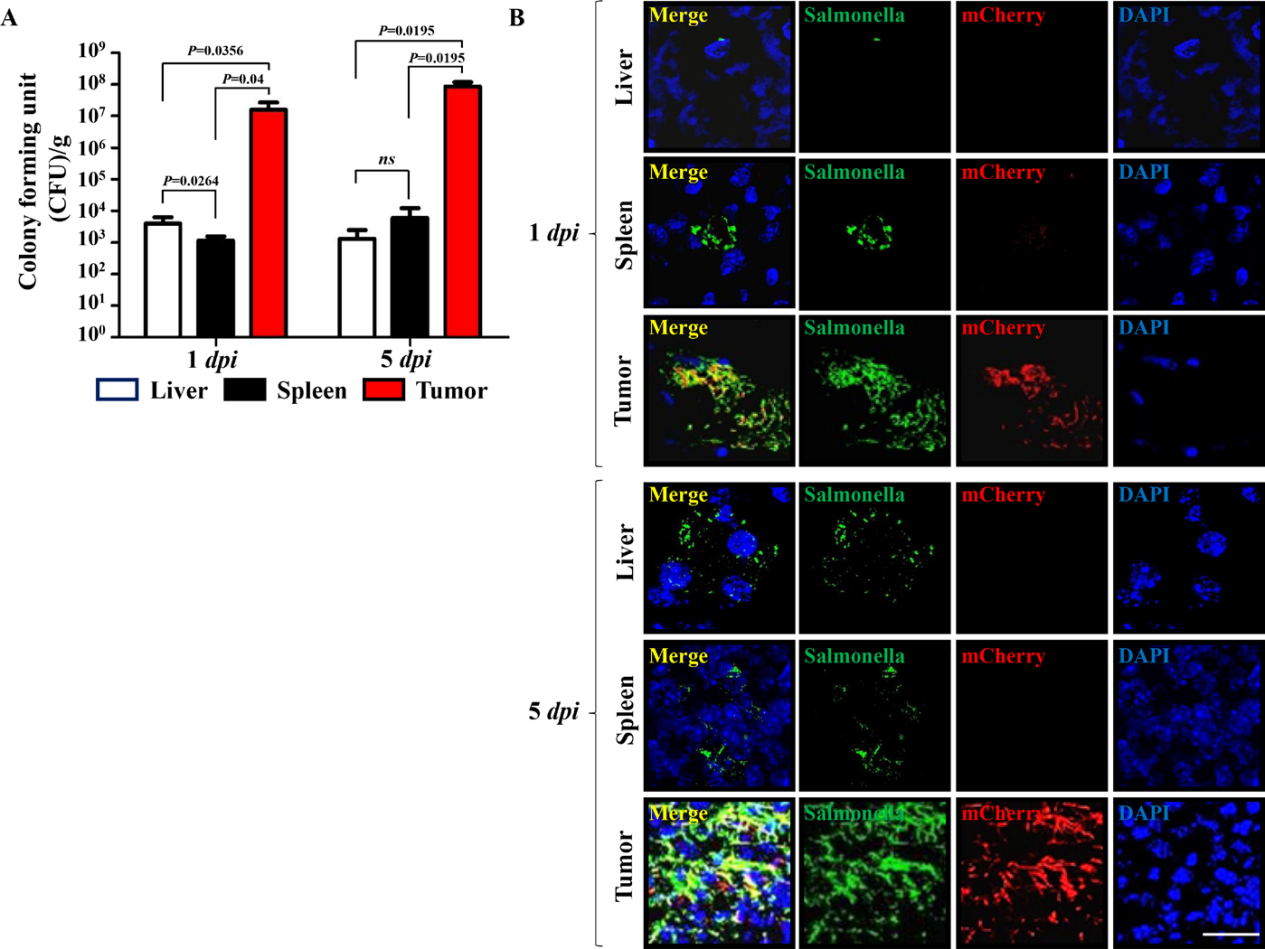
Expression of mCherry by a quorum-sensing promoter in a mouse tumor model (*n* = 3) (**A**) Bacteria in liver, spleen, and tumor tissues were counted at the indicated days by determining CFU. (**B**) Expression of mCherry from intratumoral *Salmonella* carrying pQSRI-mCherry was detected by fluorescence microscopy at the indicated dpi. Expression of mCherry (red) in tumor tissues was analyzed after staining of *Salmonella* with a specific antibody (green) and nuclei with DAPI (blue) in each sample. Scale bar, 20 µm.

Subsequently, we determined the expression and distribution of mCherry expressed by tumor-targeted *Salmonella* carrying pQSRI-mCherry in C57BL/6 mice bearing MC38 tumors (Figure 2). *Salmonella* (1 × 10^7^ CFU) were injected through the tail vein when the tumor implanted in the right lateral thigh reached ∼120 mm^3^. Mice were sacrificed on 1 and 5 dpi (days post-infection). The excised tumors were subjected to immunofluorescence staining and analyzed for *Salmonella* and mCherry expression. The number of *Salmonella* in tumor tissue was in the range of 10^7^–10^8^ CFU/g in tumor tissue on 1 dpi, and a bit higher on 5 dpi, whereas the number in liver and spleen was considerably lower, between 10^3^ and 10^4^ CFU/g tissue in liver during the same time period (Figure 2A). mCherry expression was abundant in tumor tissue at 5 dpi, mostly overlapping with *Salmonella*, but much less abundant at 1 dpi. By contrast, in the reticular endothelial system, very little mCherry was detected (Figure 2B). These results confirmed QS-dependent mCherry expression in an animal system.

### Expression and secretion of ASNase from Salmonella carrying pQSRI-ASNase

**Figure 3:**
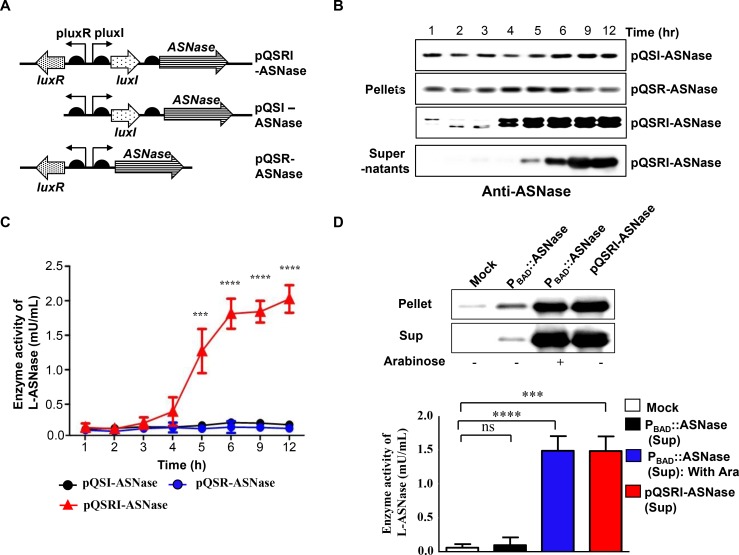
Expression of L-ASNase in ∆ppGpp Salmonella carrying pQSR-ASNase, pQSI-ASNase, or pQSRI-ASNase during growth *in vitro* (**A**) Plasmid constructs carrying ASNase under control of different combinations of QS elements, LuxR, and/or LuxI. (**B**) *Salmonella* carrying the indicated plasmids were grown in LB, and bacterial samples (pellets) were taken for analysis of L-ASNase expression by Western blotting. The last row shows L-ASNase secreted from *Salmonella* carrying pQSRI-ASNase into media (supernatant). (**C**) L-ASNase activities from *Salmonella* carrying the indicated plasmids. Asterisks (^*^) indicate significant differences vs. pQSI-mCherry (^***^P = 0.0005, ^****^P < 0.0001). (**D**) *Salmonella* were grown to stationary phase (9 hr) in LB (bacteria carrying pQSRI-ASNase) or in LB with or without 0.2% L-arabinose (bacteria carrying P_BAD_::ASNase). Equal amounts of bacterial cultures were harvested, separated into pellet and supernatant fractions, and analyzed for L-ASNase by Western blotting (upper panel) or an assay kit (supernatants only; lower panel). Asterisks (^*^) indicate significant differences vs. pBAD24 plasmid (Mock) (^***^P = 0.0004, ^****^P < 0.0001).

To develop a strain useful for treating solid malignancies using a cell density-dependent inducible QS system, the mCherry gene in the above constructs was replaced with the L-ASNase gene of *E. coli B* (BL21) [58]. To this end, we prepared three constructs, pQSRI-ASNase, pQSI-ASNase, and pQSR-ASNase (Figure 3A), and introduced them into the host bacteria. These engineered *Salmonella* were grown in LB media, and samples were taken at the indicated times. The cells were harvested by centrifugation and analyzed for L-ASNase expression by Western blotting using a specific antibody (Figure 3B). L-ASNase was induced in a cell density-dependent manner only in *Salmonella* carrying pQSRI-ASNase. The culture medium of *Salmonella* carrying pQSRI-ASNase was also harvested, and secreted protein in the medium was analyzed by Western blotting. As shown in Figure 3B (bottom row), we detected a distinct band corresponding to L-ASNase. These results indicated that L-ASNase was induced at an appropriate cell density, and that a significant fraction (∼55%) was secreted into the media, as demonstrated previously [50]. The cell density-dependent expression of L-ASNase was verified by determining the enzyme activity in *Salmonella* growing in LB medium (Figure 3C).

Previously, we engineered *Salmonella* expressing L-ASNase under the control of *P*BAD of the *E. coli* arabinose operon, which can be induced with L-arabinose (P_BAD_::ASN), and demonstrated its effects on various solid tumors in a mouse model [50]. In this study, we compared the expression of L-ASNase from pQSRI-ASNase with that from P_BAD_::ASN. For this purpose, *Salmonella* carrying pQSRI-ASNase were grown in LB medium, and cells carrying P_BAD_::ASN were grown in LB media with or without 0.2% L-arabinose. Equal amounts of bacterial cultures were harvested and separated into two fractions, bacterial pellet and media supernatant, and L-ASNase levels were analyzed by Western blotting (Figure 3D upper image). Addition of L-arabinose significantly induced L-ASNase expression in *Salmonella* carrying P_BAD_::ASNase at levels comparable to those expressed from *Salmonella* carrying pQSRI-ASNase grown in LB medium for 9 h. In both cases, more than half of L-ASNase was secreted out of the bacterial cells, which can be ascribed to the extracellular nature of the protein [59]. This was further corroborated by determination of L-ASNase enzyme activities in the same bacterial medium: the levels were very low in *Salmonella* carrying P_BAD_::ASN without induction, and the levels were higher (and comparable) in *Salmonella* carrying P_BAD_::ASN induced by L-arabinose and those carrying pQSRI-ASNase (Figure 3D lower graph).

### Antitumor effect of L-ASNase expressed by Salmonella carrying pQSRI-ASNase

**Figure 4:**
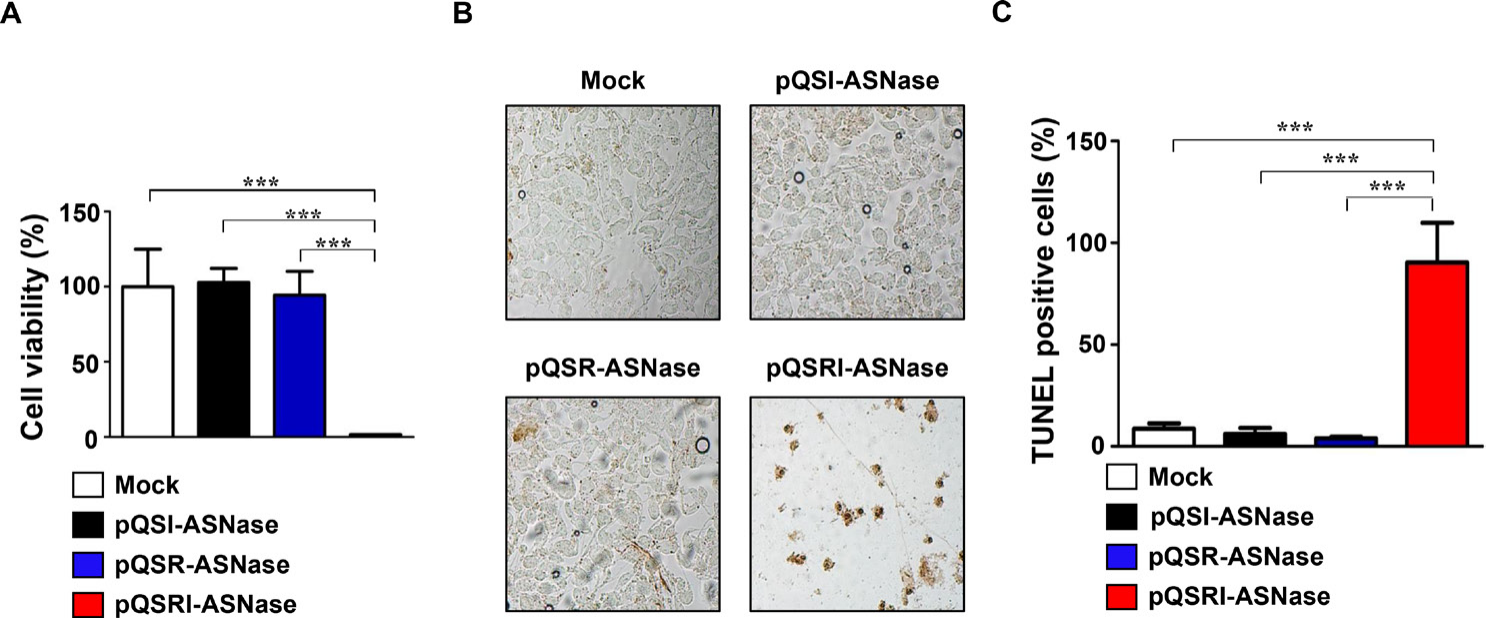
Cytotoxic effect of L-ASNase secreted from Salmonella carrying pQSRI-ASN, pQSI-ASN, or pQSR-ASNase (**A** and **B**) *Salmonella* carrying the indicated plasmids were grown in LB for 9 hr. Bacterial supernatants were separated and concentrated to 200 mg/ml. MC38 cells were treated with the supernatants for 24 hr, and cell viability was determined by MTT assay [A] and TUNEL assay [B]. Asterisks (^*^) indicate significant differences vs. Mock (^***^P = 0.0004). (**C**) Quantitation of TUNEL-positive cells. Mock was with *Salmonella* carrying vector pBAD plasmid. Asterisks (^*^) indicate significant differences compared to Mock or pQSR-ASN and pQSI-ASN (^***^P = 0.0005).

Next, we determined the antitumor effect of L-ASNase expressed and secreted by *Salmonella* carrying pQSRI-ASNase *in vitro*, using cultured mouse MC38 colon cancer cells. *Salmonella* carrying pQSR-ASNase or pQSI-ASNase were cultured for 9 h, and then the media were separated and concentrated. Concentrated media were added to cultured MC38 cells at final concentrations of 0.2 mg/ml, and then incubated for 24 h. MC38 cell viability was determined by MTT assay (Figure 4A). Media from *Salmonella* carrying pQSRI-ASNase killed almost all (99%) cells, whereas media from bacteria carrying other plasmids had little effect on viability. The cytotoxic effect of L-ASNase was also examined by TUNEL assay (Figure 4B and 4C). Over 90% of MC38 cells incubated with media from *Salmonella* carrying pQSRI-ASNase were TUNEL-positive; again, the other bacterial strains had minimal effect. Taken together, these data indicate that L-ASNase expressed and secreted under the control of the QS system is highly toxic to MC38 cancer cells *in vitro*.

**Figure 5:**
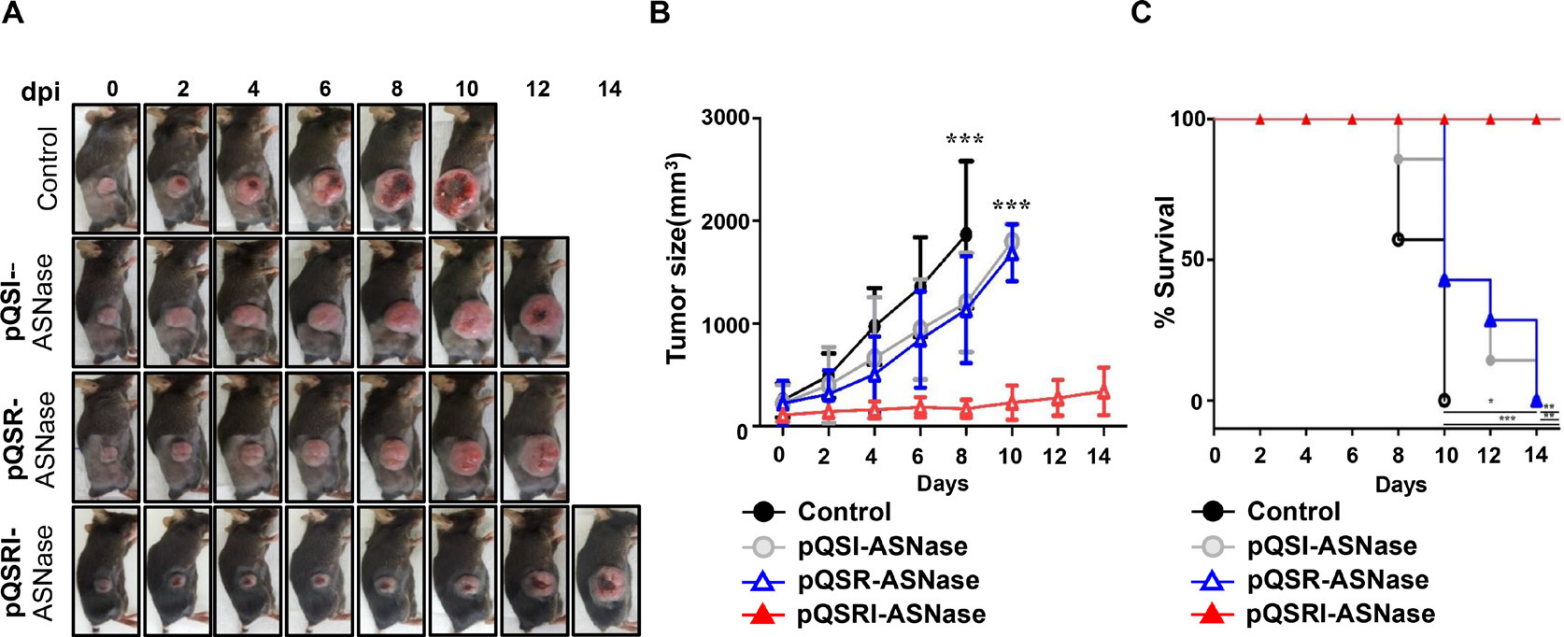
Antitumor effects of Salmonella carrying pQSRI-ASNase, pQSI-ASNase, or pQSR-ASNase *in vivo* The antitumor effect of *Salmonella* (1 × 10^7^ CFU) carrying the indicated plasmids was measured using MC38 tumors grafted into C57BL/6 mice (*n* = 7 for each treatment). Treatment with PBS was included as a negative control (*n* = 7). (**A**) Representative gross morphological changes. (**B**) Changes in tumor size after bacterial treatment. Error bars correspond to 95% confidence interval (CI). Asterisks (^*^) indicate significant differences vs. treatment with PBS (^***^P = 0.006). (**C**) Kaplan−Meier survival curves of mice bearing MC38 tumors. Asterisks (^*^) indicate significant differences vs. treatment with PBS (^*^P = 0.01, ^**^P = 0.0013, ^***^P = 0.0003).

Subsequently, we assessed the antitumor efficacy of *Salmonella* carrying pQSRI-ASNase, pQSR-ASNase, or pQSI-ASNase in C57BL/6 mice bearing MC38 tumors. *Salmonella* carrying pQSRI-ASNase had the highest antitumor efficacy (Figure 5A, 5B). *Salmonella* carrying pQSR-ASNase or pQSI-ASNase had a slightly stronger effect than the phosphate-buffered saline (PBS)-treated control, which could be ascribed to the anticancer effect of *Salmonella* itself [9, 36]. Tumors reached a mean size of 1,000 mm^3^ by day 4 following PBS treatment and by day 8 following treatment with *Salmonella* carrying either pQSR-ASNase or pQSI-ASNase. By contrast, tumors in mice treated with *Salmonella* carrying pQSRI-ASNase were less than 500 mm^3^ at the end of the experiment (day 14). Consistent with this, survival was considerably longer in animals that received *Salmonella* carrying pQSRI-ASNase than in the other groups (Figure 5C).

**Figure 6:**
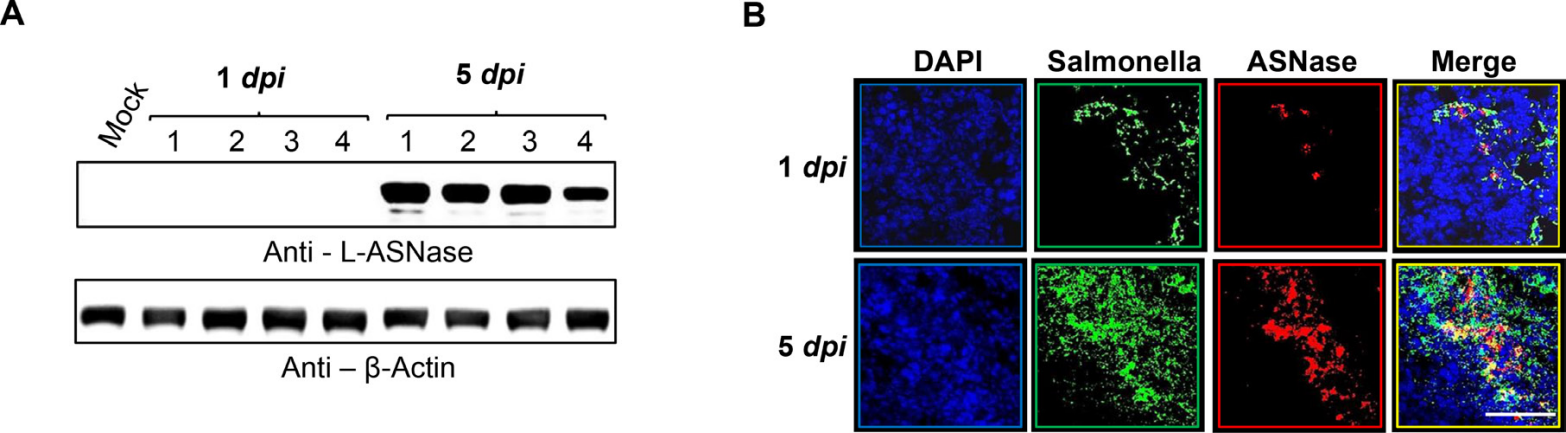
*In situ* expression of L-ASNase by quorum-sensing promoter in a mouse tumor model Expression of L-ASNase in mice treated with *Salmonella* carrying pQSRI-ASNase was analyzed at 1 and 5 dpi by Western blotting (*n* = 4 for each group, (**A**) or by immunofluorescence microscopy (80× magnification) (**B**). Green indicates *Salmonella* and red indicates L-ASNase. Scale bar, 50 µm.

Analysis of L-ASNase levels in tumor tissues from mice treated with *Salmonella* carrying pQSRI-ASNase (Figure 6) revealed little L-ASNase at 1 dpi, but a considerable level at 5 dpi, as assessed by Western blotting with a specific antibody (Figure 6A, *n* = 4) and immunofluorescence microscopy (Figure 6B). Notably, L-ASNase co-localized with *Salmonella* in a density-dependent manner, i.e., L-ASNase was detected in areas with dense *Salmonella* populations (Figure 6B). Based on these findings, we conclude that L-ASNase expressed in a bacterial density-dependent manner effectively suppressed solid tumor growth in our mouse model.

## DISCUSSION

Successful application of cancer therapy using bacteria expressing a genetically engineered anticancer protein requires that the active agent be conditionally expressed in tumor tissue, leaving the reticular endothelial system undamaged. In this study, we developed and employed an expression system that is activated in a bacterial density-dependent manner, causing the therapeutic protein under its control to be expressed only when bacterial cell density rises above a threshold level (at least 10^8^ CFU/ml, Figure 1B). Bacterial cell densities in healthy liver and spleen remained below 10^4^ CFU/g. *In vitro* experiments showed that *luxI* promoter-driven expression of the cargo gene was activated as the cultures entered stationary phase, as bacterial cell density rose above ∼10^9^ CFU/ml in liquid culture (Figure 1B). This induction was dependent on AHL produced by LuxI and its cognate binding protein LuxR. This theory was corroborated by restoration of induction by addition of exogenous AHL into the culture medium of *Salmonella* carrying pQSR-mCherry (Figure 1E).

Examination of the expression of mCherry and L-ASNase in intratumoral *Salmonella* at 1 and 5 dpi by IF microscopy (Figures 2 and 6) revealed greater abundance at 5 dpi than 1 dpi, but significantly more mCherry/L-ASNase expression at 5 dpi in the area where *Salmonella* had accumulated heavily (Figures 2B and 6). This pattern of expression suggested that AHL reached the critical concentration only in these areas of high *Salmonella* density. Consistent with this, little mCherry expression was detected in the reticular endothelial system. A similar study using *Salmonella* carrying an episomal *luxI* promoter–*luxR–GFP–luxI* construct [60] concluded that GFP expression by intratumoral *Salmonella* colonies depended on the distance between neighboring bacteria; no colonies expressed GFP when the average distance to neighbors was greater than 155 µm. Thus, this QS-dependent induction system is far superior to the *P*BAD system of *E. coli*, which provides a remote control system by which a tumor-toxic protein can be induced by i.p. administration of L-arabinose [37, 42, 50]. Consequently, sustained expression of the cargo protein by intratumoral bacteria requires repeated administration of L-arabinose. As demonstrated in this study, QS-dependent induction of the L-ASNase by intratumoral *Salmonella* provides a simple method for sustained expression of a protein drug without requiring supplementation with an exogenous inducer, and achieved notable retardation of tumor growth and delay of mortality in tumor-bearing mice (Figure 5).

## MATERIALS AND METHODS

### Bacterial strains, plasmids, and culture conditions

The *E. coli* strain used in this study for gene manipulation was XL1-Blue. The QS expression module of *Vibrio fischeri* was derived from the sequence deposited in GenBank (AF170104.1). The expression plasmid pBAD24 (Invitrogen, USA) was chosen as a scaffold for construction of the QS expression system in *Salmonella*. The *Salmonella* strain used in this study, the ppGpp-defective strain SHJ2037 (*relA::cat*, *spoT::kan*), was derived from the wild-type *Salmonella enterica* serovar *Typhimurium* 14028s, and was described previously [27, 61]. This strain is also *GLmS* ^–^, and therefore requires N-acetylglucosamine for growth unless complemented by a *GLmS* ^+^-containing plasmid [57].

Recombinant *Salmonella* were grown at 37°C in LB medium containing 1% NaCl. For solid support medium, 1.5% Bacto agar was included. All media were supplemented with an antibiotic (ampicillin, 100 µg/ml) and/or AHL (0.05–1 mM) as needed.

### Construction of recombinant plasmids with various genes under the control of the QS expression system

To express target genes under the control of the QS system, the expression vector pQS was constructed using pBAD24 as a scaffold. The primer pair LuxRF/LuxROLR (Table 1) was designed to amplify the gene encoding LuxR (accession no. AAD48473.1); the primer pair luxRI proOLF/LuxIR was used to generate a construct that fuses the *luxR–luxI* intergenic sequence with *luxI* gene (accession no. AAD48474.1). As a functional reporter, the gene encoding mCherry (Takara Bio, USA) was also amplified using primer pair mChOLF/mChR. The three amplicons were assembled by overlapping PCR, and then subcloned into the *Bam*HI/*Sal*I site of pBAD24 to yield pQSRI-mCherry (Figure 1A). The recombinant plasmid pQSI-mCherry was prepared as follows: primers luxRI proF and LuxIR were used to generate a construct that fuses the *luxR–luxI* intergenic sequence with the *luxI* gene. This DNA fragment was assembled by PCR using the same mCherry gene described above. The resultant DNA fragment was subcloned into the *Bam*HI/*Sal*I site of pBAD24. pQSR-mCherry was also constructed by overlapping PCR with two constructs, a DNA fragment containing *luxR* and the *luxR–luxI* intergenic sequence amplified by PCR with primers LuxRF and LuxRI proR, and a DNA fragment containing mCherry amplified with primers mChOL-RIF and mChR. The resultant construct was also subcloned into the *Bam*HI/*Sal*I site of pBAD24. For stable maintenance *in vivo* without exogenous inducer, the *glmS* gene was amplified with primers *glmSF* and *glmSR* primers from genomic DNA of *Salmonella* and inserted into the vector at the *Cla*I site.

To evaluate these constructs as a genetically encoded drug expression system *in vivo*, the three plasmids pQSRI-ASNase, pQSI-ASNase, and pQSR-ASNase, in which the mCherry gene was replaced with the gene encoding ASNase, were constructed using the procedure described above (Figure 3A). These constructions used DNA fragments amplified with primer pairs ASN OLF/ASN R and ASN OL-RIF/ASN R. All constructs were confirmed by DNA sequencing and functionally tested in both *E. coli* XL1-Blue and DppGpp *S. typhimurium*.

### Preparation of L-ASNase

To monitor the intracellular expression level of L-ASNase, recombinant bacteria were harvested by centrifugation at 5,000 × *g* for 5 min and lysed by sonication in lysis buffer (10 mM lysozyme, 10% sodium dodecyl sulfate). The resultant solution was further centrifuged at 13,000 × *g* for 30 min, and the supernatant was collected, filtered (0.22 µm pore size), and concentrated using a Centricon device (Amicon^®^ Ultra, 10K pore filter, Millipore). The proteins were precipitated with 10% trichloroacetic acid (TCA) for 1 hr at 4°C, and the pellets were re-dissolved in PBS for analysis of secreted L-ASNase.

### Cell culture and viability assay

MC38 cells were grown in high-glucose Dulbecco’s modified Eagle medium (DMEM) containing 10% fetal bovine serum (FBS) and 1% penicillin–streptomycin.

Cell viability was assessed using the 3-(4,5-dimethylthiazol-2-yl)-2,5-diphenyltetrazolium bromide (MTT) colorimetric assay. Briefly, 100 μl cell suspension was inoculated in each well of a 96-well plate at a density of 2 × 10^4^ cells/well. After 24 hr of culture, the medium was removed by aspiration and replaced with 100 μl experimental medium, and the cells were cultured for an additional 24 hr. MTT solution (10 μl) was added to each well, and the plates were incubated in the dark for 4 hr at 37°C. The number of remaining viable cells (i.e., cells with MTT dye) was determined by measuring the optical density at 490 nm in an enzyme-linked immunosorbent assay reader.

### TUNEL assays

*Salmonella* carrying pQSRI-ASNase, pQSI-ASNase, or pQSR-ASNase were cultured in LB for 24 hr. Bacterial supernatants were harvested as described above. MC38 cells were incubated with the bacterial supernatants (0.2 mg/ml) for 24 hr, fixed in 4% paraformaldehyde (PFA), and analyzed using the DeadEnd colorimetric TUNEL analysis kit (Promega, Madison, WI, USA).

### Immunofluorescence staining

Tumor tissues were fixed overnight at room temperature with 4% PFA in PBS. Samples were embedded and frozen in optical Optimal Cutting Temperature compound (OCT, Tissue-Tek), and then sliced into 6 mm sections using a microtome-cryostat. Tissue sections were collected on aminopropyl triethoxysilane-coated slides. The slides were washed in PBS (pH 7.4) and incubated with primary antibody, rabbit anti-asparaginase (1:100, Abcam, ab28364), and mouse anti-*Salmonella* (1:100, Abcam, ab8274) overnight at 4°C. Subsequently, Alexa Fluor 488-conjugated goat anti-rabbit (1:100) and Alexa Fluor 568-conjugated goat anti-mouse (1:100) antibodies were used as secondary antibodies. After staining of nuclei with DAPI/Antifade (1:200, Invitrogen), the samples were mounted.

### Western analysis

Mammalian cell lines and tumor tissues were lysed by sonication in RIPA buffer (50 mM Tris-HCl [pH 7.5], 150 mM NaCl, 1% NP40, 0.5% sodium deoxycholate, and 0.1% SDS) containing protease inhibitors (protease inhibitor mixture; Roche Applied Science). Tumor tissues were extracted, homogenized with a motor-driven tissue homogenizer (Ika-Werke, Staufen, Germany), and separated into supernatant and pellet by centrifugation (5,000 × *g*, 5 min). Pellets were lysed by sonication as described above. The supernatants were treated with 10% TCA (1 hr, 4°C) to precipitate proteins. For Western blot analysis, protein samples were boiled for 5 min, separated by 10% SDS–polyacrylamide gel electrophoresis (PAGE), and transferred to nitrocellulose membrane (Amersham Biosciences, Buckinghamshire, UK). The membrane was blocked with 5% skim milk, probed at 4°C overnight with rabbit anti- mcherry antibody (1:1000; abcam), rabbit anti-asparaginase antibody (1:1,000; Abcam) or mouse anti-b-Actin antibody (1:5,000, Santa Cruz Biotechnology), and then incubated with anti-mouse or anti-rabbit IgG linked to horseradish peroxidase (Sigma-Aldrich, UK) for 1 hr, respectively. Bound proteins were visualized using the ECL kit (Amersham Biosciences).

### Animal experiments

Five- to six-week-old male mice (20–30 g body weight) were purchased from Samtako (Korea). When the tumor reached ∼120 mm^3^, 1 × 10^7^ CFU ∆ppGpp *S. typhimurium* transformed with the indicated plasmids were suspended in PBS and injected through the tail vein. All animal care, experiments, and euthanasia were performed in accordance with protocols approved by the Chonnam National University Animal Research Committee. Animals were anesthetized with isoflurane (2%) for imaging or a mixture of ketamine (200 mg kg^-1^) and xylazine (10 mg kg^-1^) for surgery. Mice carrying subcutaneous tumors were generated as follows: tumor cells cultured *in vitro* were harvested, suspended in 100 µl PBS, and injected subcutaneously into the right thigh (1 × 10^6^ cells for MC38). Tumor volumes (mm^3^) were estimated using the formula (L × H × W)/2, where L is the length, W is the width, and H is the height of the tumor in millimeters.

### L-ASNase activity assay

Bacterial supernatants containing L-ASNase were prepared in concentrated forms for the enzymatic assay. Tumor tissue samples were extracted with four volumes of assay buffer from the Asparaginase Activity Assay kit (Abcam, ab107922), and insoluble materials were removed by centrifugation. Levels of L-ASNase enzymatic activity in bacterial supernatants (100 µg) and tumor tissues (100 µg) were assessed using the Asparaginase Activity Assay kit.

### Statistical analysis

Statistical analysis was performed using the SPSS 18.0 statistical package (SPSS Inc., Chicago, IL, USA). The two-tailed Student’s *t-*test was used to assess the statistical significance of tumor growth and differences in survival between treatment groups. *P* < 0.05 was considered statistically significant.

## References

[1] Min JJ, Nguyen VH, Kim HJ, Hong Y, Choy HE (2008). Quantitative bioluminescence imaging of tumor-targeting bacteria in living animals. Nat Protoc.

[2] Zheng JH, Nguyen VH, Jiang SN, Park SH, Tan W, Hong SH, Shin MG, Chung IJ, Hong Y, Bom HS, Choy HE, Lee SE, Rhee JH (2017). Two-step enhanced cancer immunotherapy with engineered Salmonella typhimurium secreting heterologous flagellin. Sci Transl Med.

[3] Cheong I, Huang X, Bettegowda C, Diaz LA, Kinzler KW, Zhou S, Vogelstein B (2006). A bacterial protein enhances the release and efficacy of liposomal cancer drugs. Science.

[4] Mason NJ, Gnanandarajah JS, Engiles JB, Gray F, Laughlin D, Gaurnier-Hausser A, Wallecha A, Huebner M, Paterson Y (2016). Immunotherapy with a HER2-Targeting Listeria Induces HER2-Specific Immunity and Demonstrates Potential Therapeutic Effects in a Phase I Trial in Canine Osteosarcoma. Clin Cancer Res.

[5] Leschner S, Weiss S (2010). Salmonella-allies in the fight against cancer. J Mol Med (Berl).

[6] Toso JF, Gill VJ, Hwu P, Marincola FM, Restifo NP, Schwartzentruber DJ, Sherry RM, Topalian SL, Yang JC, Stock F, Freezer LJ, Morton KE, Seipp C (2002). Phase I study of the intravenous administration of attenuated Salmonella typhimurium to patients with metastatic melanoma. J Clin Oncol.

[7] Heimann DM, Rosenberg SA (2003). Continuous intravenous administration of live genetically modified salmonella typhimurium in patients with metastatic melanoma. J Immunother.

[8] Nemunaitis J, Cunningham C, Senzer N, Kuhn J, Cramm J, Litz C, Cavagnolo R, Cahill A, Clairmont C, Sznol M (2003). Pilot trial of genetically modified, attenuated Salmonella expressing the E. coli cytosine deaminase gene in refractory cancer patients. Cancer Gene Ther.

[9] Zhao M, Yang M, Li XM, Jiang P, Baranov E, Li S, Xu M, Penman S, Hoffman RM (2005). Tumor-targeting bacterial therapy with amino acid auxotrophs of GFP-expressing Salmonella typhimurium. Proc Natl Acad Sci U S A.

[10] Hayashi K, Zhao M, Yamauchi K, Yamamoto N, Tsuchiya H, Tomita K, Hoffman RM (2009). Cancer metastasis directly eradicated by targeted therapy with a modified Salmonella typhimurium. J Cell Biochem.

[11] Nagakura C, Hayashi K, Zhao M, Yamauchi K, Yamamoto N, Tsuchiya H, Tomita K, Bouvet M, Hoffman RM (2009). Efficacy of a genetically-modified Salmonella typhimurium in an orthotopic human pancreatic cancer in nude mice. Anticancer Res.

[12] Yam C, Zhao M, Hayashi K, Ma H, Kishimoto H, McElroy M, Bouvet M, Hoffman RM (2010). Monotherapy with a tumor-targeting mutant of S. typhimurium inhibits liver metastasis in a mouse model of pancreatic cancer. J Surg Res.

[13] Zhao M, Geller J, Ma H, Yang M, Penman S, Hoffman RM (2007). Monotherapy with a tumor-targeting mutant of Salmonella typhimurium cures orthotopic metastatic mouse models of human prostate cancer. Proc Natl Acad Sci U S A.

[14] Zhao M, Yang M, Ma H, Li X, Tan X, Li S, Yang Z, Hoffman RM (2006). Targeted therapy with a Salmonella typhimurium leucine-arginine auxotroph cures orthotopic human breast tumors in nude mice. Cancer Res.

[15] Hiroshima Y, Zhao M, Maawy A, Zhang Y, Katz MH, Fleming JB, Uehara F, Miwa S, Yano S, Momiyama M, Suetsugu A, Chishima T, Tanaka K (2014). Efficacy of Salmonella typhimurium A1-R versus chemotherapy on a pancreatic cancer patient-derived orthotopic xenograft (PDOX). J Cell Biochem.

[16] Hiroshima Y, Zhang Y, Murakami T, Maawy A, Miwa S, Yamamoto M, Yano S, Sato S, Momiyama M, Mori R, Matsuyama R, Chishima T, Tanaka K (2014). Efficacy of tumor-targeting Salmonella typhimurium A1-R in combination with anti-angiogenesis therapy on a pancreatic cancer patient-derived orthotopic xenograft (PDOX) and cell line mouse models. Oncotarget.

[17] Hiroshima Y, Zhao M, Zhang Y, Zhang N, Maawy A, Murakami T, Mii S, Uehara F, Yamamoto M, Miwa S, Yano S, Momiyama M, Mori R (2015). Tumor-Targeting Salmonella typhimurium A1-R Arrests a Chemo-Resistant Patient Soft-Tissue Sarcoma in Nude Mice. PLoS One.

[18] Murakami T, DeLong J, Eilber FC, Zhao M, Zhang Y, Zhang N, Singh A, Russell T, Deng S, Reynoso J, Quan C, Hiroshima Y, Matsuyama R (2016). Tumor-targeting Salmonella typhimurium A1-R in combination with doxorubicin eradicate soft tissue sarcoma in a patient-derived orthotopic xenograft (PDOX) model. Oncotarget.

[19] Kiyuna T, Murakami T, Tome Y, Kawaguchi K, Igarashi K, Zhang Y, Zhao M, Li Y, Bouvet M, Kanaya F, Singh A, Dry S, Eilber FC, Hoffman RM (2016). High efficacy of tumor-targeting Salmonella typhimurium A1-R on a doxorubicin- and dactolisib-resistant follicular dendritic-cell sarcoma in a patient-derived orthotopic xenograft PDOX nude mouse model. Oncotarget.

[20] Yamamoto M, Zhao M, Hiroshima Y, Zhang Y, Shurell E, Eilber FC, Bouvet M, Noda M, Hoffman RM (2016). Efficacy of Tumor-Targeting Salmonella A1-R on a Melanoma Patient-Derived Orthotopic Xenograft (PDOX) Nude-Mouse Model. PLoS One.

[21] Kawaguchi K, Igarashi K, Murakami T, Chmielowski B, Kiyuna T, Zhao M, Zhang Y, Singh A, Unno M, Nelson SD, Russell TA, Dry SM, Li Y (2016). Tumor-targeting Salmonella typhimurium A1-R combined with temozolomide regresses malignant melanoma with a BRAF-V600E mutation in a patient-derived orthotopic xenograft (PDOX) model. Oncotarget.

[22] Igarashi K, Kawaguchi K, Murakami T, Kiyuna T, Miyake K, Nelson SD, Dry SM, Li Y, Yanagawa J, Russell TA, Singh AS, Yamamoto N, Hayashi K (2017). Intra-arterial administration of tumor-targeting Salmonella typhimurium A1-R regresses a cisplatin-resistant relapsed osteosarcoma in a patient-derived orthotopic xenograft (PDOX) mouse model. Cell Cycle.

[23] Kawaguchi K, Igarashi K, Murakami T, Zhao M, Zhang Y, Chmielowski B, Kiyuna T, Nelson SD, Russell TA, Dry SM, Li Y, Unno M, Eilber FC (2017). Tumor-Targeting Salmonella typhimurium A1-R Sensitizes Melanoma With a BRAF-V600E Mutation to Vemurafenib in a Patient-Derived Orthotopic Xenograft (PDOX) Nude Mouse Model. J Cell Biochem.

[24] Kawaguchi K, Igarashi K, Murakami T, Kiyuna T, Zhao M, Zhang Y, Nelson SD, Russell TA, Dry SM, Singh AS, Chmielowski B, Li Y, Unno M (2017). Salmonella typhimurium A1-R targeting of a chemotherapy-resistant BRAF-V600E melanoma in a patient-derived orthotopic xenograft (PDOX) model is enhanced in combination with either vemurafenib or temozolomide. Cell Cycle.

[25] Yano S, Zhang Y, Zhao M, Hiroshima Y, Miwa S, Uehara F, Kishimoto H, Tazawa H, Bouvet M, Fujiwara T, Hoffman RM (2014). Tumor-targeting Salmonella typhimurium A1-R decoys quiescent cancer cells to cycle as visualized by FUCCI imaging and become sensitive to chemotherapy. Cell Cycle.

[26] Yano S, Takehara K, Zhao M, Tan Y, Han Q, Li S, Bouvet M, Fujiwara T, Hoffman RM (2016). Tumor-specific cell-cycle decoy by Salmonella typhimurium A1-R combined with tumor-selective cell-cycle trap by methioninase overcome tumor intrinsic chemoresistance as visualized by FUCCI imaging. Cell Cycle.

[27] Song M, Kim HJ, Ryu S, Yoon H, Yun J, Choy HE (2010). ppGpp-mediated stationary phase induction of the genes encoded by horizontally acquired pathogenicity islands and cob/pdu locus in Salmonella enterica serovar Typhimurium. J Microbiol.

[28] Song M, Kim HJ, Kim EY, Shin M, Lee HC, Hong Y, Rhee JH, Yoon H, Ryu S, Lim S, Choy HE (2004). ppGpp-dependent stationary phase induction of genes on Salmonella pathogenicity island 1. J Biol Chem.

[29] Jeong JH, Song M, Park SI, Cho KO, Rhee JH, Choy HE (2008). Salmonella enterica serovar gallinarum requires ppGpp for internalization and survival in animal cells. J Bacteriol.

[30] Nguyen VH, Kim HS, Ha JM, Hong Y, Choy HE, Min JJ (2010). Genetically engineered Salmonella typhimurium as an imageable therapeutic probe for cancer. Cancer Res.

[31] Le UN, Kim HS, Kwon JS, Kim MY, Nguyen VH, Jiang SN, Lee BI, Hong Y, Shin MG, Rhee JH, Bom HS, Ahn Y, Gambhir SS (2011). Engineering and visualization of bacteria for targeting infarcted myocardium. Mol Ther.

[32] Kim JE, Phan TX, Nguyen VH, Dinh-Vu HV, Zheng JH, Yun M, Park SG, Hong Y, Choy HE, Szardenings M, Hwang W, Park JA, Park S (2015). Salmonella typhimurium Suppresses Tumor Growth via the Pro-Inflammatory Cytokine Interleukin-1beta. Theranostics.

[33] Phan TX, Nguyen VH, Duong MT, Hong Y, Choy HE, Min JJ (2015). Activation of inflammasome by attenuated Salmonella typhimurium in bacteria-mediated cancer therapy. Microbiol Immunol.

[34] Chorobik P, Czaplicki D, Ossysek K, Bereta J (2013). Salmonella and cancer: from pathogens to therapeutics. Acta Biochim Pol.

[35] Min JJ, Kim HJ, Park JH, Moon S, Jeong JH, Hong YJ, Cho KO, Nam JH, Kim N, Park YK, Bom HS, Rhee JH, Choy HE (2008). Noninvasive real-time imaging of tumors and metastases using tumor-targeting light-emitting Escherichia coli. Mol Imaging Biol.

[36] Pawelek JM, Low KB, Bermudes D (1997). Tumor-targeted Salmonella as a novel anticancer vector. Cancer Res.

[37] Agrawal N, Bettegowda C, Cheong I, Geschwind JF, Drake CG, Hipkiss EL, Tatsumi M, Dang LH, Diaz LA, Pomper M, Abusedera M, Wahl RL, Kinzler KW (2004). Bacteriolytic therapy can generate a potent immune response against experimental tumors. Proc Natl Acad Sci U S A.

[38] Crull K, Bumann D, Weiss S (2011). Influence of infection route and virulence factors on colonization of solid tumors by Salmonella enterica serovar Typhimurium. FEMS Immunol Med Microbiol.

[39] Loessner H, Endmann A, Leschner S, Westphal K, Rohde M, Miloud T, Hammerling G, Neuhaus K, Weiss S (2007). Remote control of tumour-targeted Salmonella enterica serovar Typhimurium by the use of L-arabinose as inducer of bacterial gene expression *in vivo*. Cell Microbiol.

[40] Forbes NS (2010). Engineering the perfect (bacterial) cancer therapy. Nat Rev Cancer.

[41] Ryan RM, Green J, Williams PJ, Tazzyman S, Hunt S, Harmey JH, Kehoe SC, Lewis CE (2009). Bacterial delivery of a novel cytolysin to hypoxic areas of solid tumors. Gene Ther.

[42] Jiang SN, Park SH, Lee HJ, Zheng JH, Kim HS, Bom HS, Hong Y, Szardenings M, Shin MG, Kim SC, Ntziachristos V, Choy HE, Min JJ (2013). Engineering of bacteria for the visualization of targeted delivery of a cytolytic anticancer agent. Mol Ther.

[43] Jeong JH, Kim K, Lim D, Jeong K, Hong Y, Nguyen VH, Kim TH, Ryu S, Lim JA, Kim JI, Kim GJ, Kim SC, Min JJ (2014). Anti-tumoral effect of the mitochondrial target domain of Noxa delivered by an engineered Salmonella typhimurium. PLoS One.

[44] Wriston JC, Yellin TO (1973). L-asparaginase: a review. Adv Enzymol Relat Areas Mol Biol.

[45] Willems L, Jacque N, Jacquel A, Neveux N, Maciel TT, Lambert M, Schmitt A, Poulain L, Green AS, Uzunov M, Kosmider O, Radford-Weiss I, Moura IC (2013). Inhibiting glutamine uptake represents an attractive new strategy for treating acute myeloid leukemia. Blood.

[46] Ueno T, Ohtawa K, Mitsui K, Kodera Y, Hiroto M, Matsushima A, Inada Y, Nishimura H (1997). Cell cycle arrest and apoptosis of leukemia cells induced by L-asparaginase. Leukemia.

[47] Cohen HJ (2003). Treatment of Acute Leukemias: New Directions for Clinical Research. N Engl J Med.

[48] Hays JL, Kim G, Walker A, Annunziata CM, Lee JM, Squires J, Houston N, Steinberg SM, Kohn EC (2013). A phase II clinical trial of polyethylene glycol-conjugated L-asparaginase in patients with advanced ovarian cancer: Early closure for safety. Mol Clin Oncol.

[49] Muller HJ, Boos J (1998). Use of L-asparaginase in childhood ALL. Crit Rev Oncol Hematol.

[50] Kim K, Jeong JH, Lim D, Hong Y, Lim HJ, Kim GJ, Shin SR, Lee JJ, Yun M, Harris RA, Min JJ, Choy HE (2015). L-Asparaginase delivered by Salmonella typhimurium suppresses solid tumors. Mol Ther Oncolytics.

[51] Balasubramanian MN, Butterworth EA, Kilberg MS (2013). Asparagine synthetase: regulation by cell stress and involvement in tumor biology. Am J Physiol Endocrinol Metab.

[52] Engebrecht J, Silverman M (1984). Identification of genes and gene products necessary for bacterial bioluminescence. Proc Natl Acad Sci U S A.

[53] Eberhard A, Burlingame AL, Eberhard C, Kenyon GL, Nealson KH, Oppenheimer NJ (1981). Structural identification of autoinducer of Photobacterium fischeri luciferase. Biochemistry.

[54] Engebrecht J, Nealson K, Silverman M (1983). Bacterial bioluminescence: isolation and genetic analysis of functions from Vibrio fischeri. Cell.

[55] Kaplan HB, Greenberg EP (1985). Diffusion of autoinducer is involved in regulation of the Vibrio fischeri luminescence system. J Bacteriol.

[56] Stevens AM, Dolan KM, Greenberg EP (1994). Synergistic binding of the Vibrio fischeri LuxR transcriptional activator domain and RNA polymerase to the lux promoter region. Proc Natl Acad Sci U S A.

[57] Kim K, Jeong JH, Lim D, Hong Y, Yun M, Min JJ, Kwak SJ, Choy HE (2013). A novel balanced-lethal host-vector system based on glmS. PLoS One.

[58] Daegelen P, Studier FW, Lenski RE, Cure S, Kim JF (2009). Tracing ancestors and relatives of Escherichia coli B, and the derivation of B strains REL606 and BL21(DE3). J Mol Biol.

[59] Jennings MP, Beacham IR (1990). Analysis of the Escherichia coli gene encoding L-asparaginase II, ansB, and its regulation by cyclic AMP receptor and FNR proteins. J Bacteriol.

[60] Swofford CA, Van Dessel N, Forbes NS (2015). Quorum-sensing Salmonella selectively trigger protein expression within tumors. Proc Natl Acad Sci U S A.

[61] Flentie K, Kocher B, Gammon ST, Novack DV, McKinney JS, Piwnica-Worms D (2012). A bioluminescent transposon reporter-trap identifies tumor-specific microenvironment-induced promoters in Salmonella for conditional bacterial-based tumor therapy. Cancer Discov.

